# Long noncoding RNA taurine-up regulated gene 1 for the prognosis of osteosarcoma: A protocol for meta-analysis and bioinformatics analysis

**DOI:** 10.1097/MD.0000000000026182

**Published:** 2021-06-18

**Authors:** Zheng Ren, Chenyang Li, Yuling Gan, Xiuxin Liu, Fudong Liang

**Affiliations:** a Department of Orthopedic Trauma, The Sixth Affiliated Hospital of Xinjiang Medical University; b Department of Hand Foot Microsurgery, The Third People's Hospital of Xinjiang Uygur Autonomous Region, Urumqi, Xin Jiang Province; c Department of Orthopedics and Soft Surgery, Gansu Cancer Hospital, Lanzhou, Gansu Province, China.

**Keywords:** bioinformatics, meta-analysis, osteosarcoma, prognosis, protocol, taurine-up regulated gene 1

## Abstract

**Background:**

In recent years, a variety of long noncoding RNA (lncRNA) has been confirmed to be involved in the initiation and progression of osteosarcoma. Taurine-up regulated gene 1 (TUG1) plays an important role in the formation, invasion, and metastasis of osteosarcoma. Therefore, perhaps TUG1 is a potential biomarker for the prognosis of patients suffering from osteosarcoma. In this study, meta-analysis and bioinformatics were adopted to further explore the effects of TUG1 on the prognosis of patients with osteosarcoma and its potential molecular mechanism.

**Methods:**

Embase, PubMed, Sinomed, Web of Science, Cochrane Library, China National Knowledge Infrastructure, Wanfang database, and Vip Journal Integration Platform were searched from inception to May 2021. The relationship between TUG1 expression and survival outcome was estimated by hazard ratio (HRs) and 95% confidence interval (CIs). Meta-analysis was conducted on the Stata 16.0. The differential expression of TUG1 in osteosarcoma was analyzed by using UALCAN database, and the survival of TUG1 was analyzed as well. The target genes of TUG1 were predicted by RegRNA2.0 biology software, HMDD, targetscan and microTCDS, and TUG1-micoRNAs-mRNAs regulatory network was constructed. The predicted target genes obtained GeneOntology (GO) analysis and Kyoto Encyclopedia of Genes and Genomes (KEGG) signal transduction pathway enrichment analysis using FunRich platform.

**Results:**

The results of this meta-analysis would be submitted to peer-reviewed journals for publication.

**Conclusion:**

This study will provide evidence-based medical evidence for the relationship between TUG1 and the prognosis of osteosarcoma. Furthermore, bioinformatics analysis will provide ideas for the exploration on osteosarcoma mechanism.

**Ethics and dissemination:**

The private information from individuals will not be published. This systematic review also should not damage participants’ rights. Ethical approval is not available. The results will be published in a peer-reviewed journal or disseminated in relevant conferences.

**OSF registration number:**

DOI 10.17605/OSF.IO/CW4BF.

## 1 Introduction

As the most common primary malignant bone tumor in children and adolescents, osteosarcoma occurs in the metaphysis of long bone, especially in the distal femur and proximal tibia.^[1,2]^ Rapid growth and early metastasis are the main factors for poor prognosis of osteosarcoma.^[3,4]^ The prognosis of patients has been improved by standardizing multidrug regimens of neoadjuvant chemotherapy and surgery, while metastasis occurs early, with high malignant degree and bad therapeutic effects,^[5]^ because the early clinical symptoms are not obvious. In recent decades, little progress has been made in the treatment of osteosarcoma, and the 5-year survival rate is only 55% to 65%.^[6]^ The 2-year survival rate of patients with lung metastasis is <25%.^[6]^ The survival time enters a plateau after treatment. It is difficult for traditional treatment scheme to achieve a breakthrough. Therefore, further understanding the occurrence and development of osteosarcoma and the exact mechanism of recurrence and metastasis, and exploring the molecular markers for early diagnosis of osteosarcoma and new therapeutic targets have become hot spots in this field.

Long-stranded non-coding RNA (lncRNA) is a class of RNA molecules with >200 bp transcripts and it cannot encode proteins.^[7]^ At first, lncRNA was regarded as “noise” in the process of transcription. With the deepening of the research, researchers have discovered that lncRNA is involved in tumor cell proliferation, apoptosis, invasion and metastasis, and other biological processes.^[8]^ Therefore, lncRNA may play an important role in the occurrence and development of malignant tumors.^[9]^

Taurine up-regulated gene 1 (TUG1), located on chromosome 22 with a length of about 7542 bp, was first found in taurine-induced retinal cells.^[10]^ In recent years, more and more studies have confirmed that TUG1 is important in the formation, invasion, and metastasis of osteosarcoma.^[11–14]^ Apart from it, studies have revealed that there is a certain relationship between TUG1 and the prognosis of patients with osteosarcoma.^[13,15–18]^ However, there is no systematic review and meta-analysis to report the specific role of TUG1 in the pathogenesis of osteosarcoma and the value of differential expression of TUG1 in the prognosis of patients with osteosarcoma. In this study, meta-analysis and bioinformatics were adopted to analyze the mechanism of the occurrence and development of osteosarcoma, so as to explore the value of TUG1 in evaluating the prognosis of patients with osteosarcoma.

## 2 Methods

### 2.1 Protocol register

This protocol of systematic review and meta-analysis has been drafted under the guidance of the preferred reporting items for systematic reviews and meta-analyses protocols. Moreover, it has been registered on open science framework (Registration number: DOI 10.17605/OSF.IO/CW4BF).

### 2.2 Ethics

Since this is a protocol without patient recruitment and personal information collection, the approval of the ethics committee is not required.

### 2.3 Inclusion criteria

(1) Patients who were clearly diagnosed as osteosarcoma by pathological examination;
(2) A cohort study that studied the correlation between TUG1 and prognosis of osteosarcoma;
(3) The expression level of TUG1 in each study was divided into 2 levels based on cut-off value: high and low;
(4) Follow-up data included overall survival (OS), progression-free survival or disease-free survival;
(5) Language was limited to Chinese and English;
(6) Sufficient data were included to extract or calculate the hazard ratio (HR).

### 2.4 Exclusion criteria

(1) Studies published repeatedly;
(2) Studies on literatures with incomplete abstract or data, or available data after contacting the author;
(3) Conference summaries, comments, abstracts, reviews, case reports, animal experiments, etc.

### 2.5 Search strategy

Embase, PubMed, Sinomed, Web of Science, Cochrane Library, China National Knowledge Infrastructure, Wanfang database, and Vip Journal Integration Platform were searched from inception to May 2021 by searching subject words and keywords. The search strategy for PubMed is shown in Table 1.

**Table 1:** Search strategy in PubMed database.

Number	Search terms
#1	Osteosarcoma[MeSH]
#2	Sarcoma, Osteogenic[Title/Abstract]
#3	Osteogenic Sarcoma[Title/Abstract]
#4	Osteosarcoma Tumor[Title/Abstract]
#5	Osteogenic Sarcomas[Title/Abstract]
#6	Osteosarcoma Tumors[Title/Abstract]
#7	Osteosarcomas[Title/Abstract]
#8	Sarcomas, Osteogenic[Title/Abstract]
#9	Tumor, Osteosarcoma[Title/Abstract]
#10	Tumors, Osteosarcoma[Title/Abstract]
#11	or/1–10
#12	Taurine-up regulated gene 1[Title/Abstract]
#13	TUG1[Title/Abstract]
#14	or/12–13
#15	Prognos^∗^[Title/Abstract]
#16	Survival [Title/Abstract]
#17	or/15–16
#18	#11 and #14 and #17

### 2.6 Data screening and extraction

**Figure 1:**
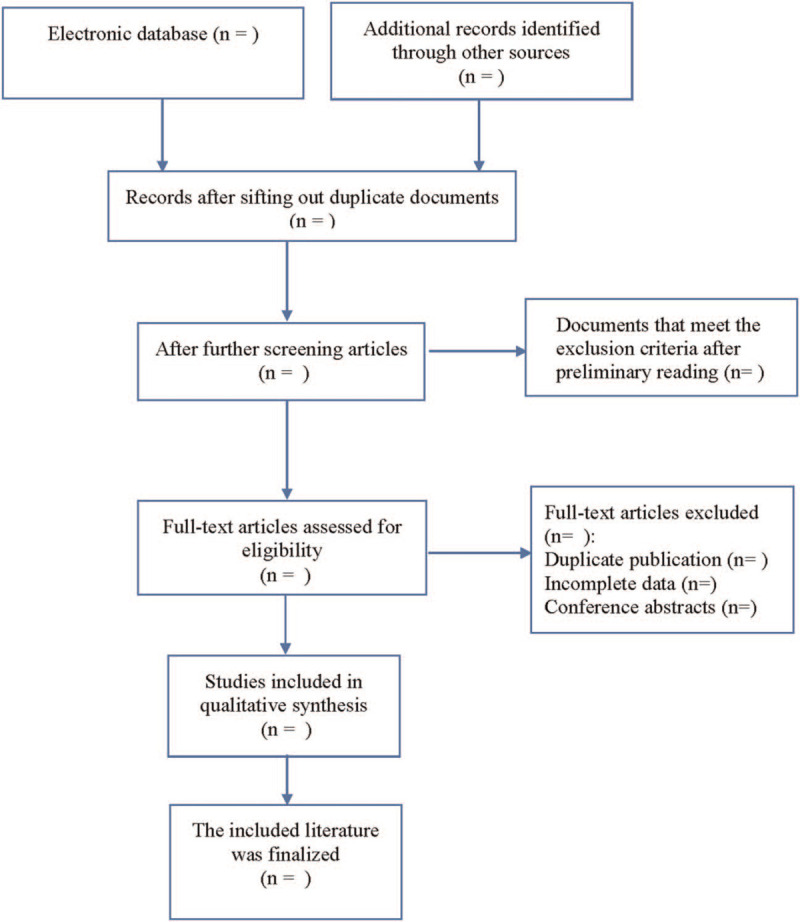
Flow diagram of study selection process.

The literature screening process is displayed in Fig. 1. According to the literature inclusion and exclusion criteria, all literatures were screened and relevant data were extracted. The data extracted from the literature include: first author, the year of publication, the country of publication, the source of the study population, the number of cases, detection methods, prognostic indicators, age, sex, degree of tumor differentiation, tumor diameter, depth of tumor invasion, lymph node metastasis, distant metastasis, TNM stage, and so on.

### 2.7 Literature quality assessment

The Newcastle-Ottawa-Scale (NOS) was used to evaluate the quality of the literatures included in the meta-analysis.^[19,20]^ If the NOS score of the literature is ≥6, it can be considered as high quality.

### 2.8 Dealing with missing data

If the data of the required study are incomplete or not reported in the study, the researcher will contact the first author or other authors by phone or email. If the required data are not available, we will use descriptive analysis instead of meta-analysis or exclude these studies if necessary.

### 2.9 Statistical analysis

#### 2.9.1 Data analysis and processing

This meta-analysis was performed using STATA 16.0 (STATA Corporation, College Station, TX). HR and the corresponding 95% CI or *P* value were chosen to evaluate the prognosis. Heterogeneity in included studies was assessed using Cochran *Q* test and Higgins *I*^2^. If *P* ≥ .1, and *I*^2^ ≤ 50%, there was low inter-study heterogeneity, and the fixed-effect model was adopted. If *P* < .1, and *I*^2^ > 50%, it indicated inter-study heterogeneity and should explore the source of heterogeneity and the random-effects model was adopted.

#### 2.9.2 Subgroup analysis

In this study, a subgroup analysis will be conducted based on the cut-off value of TUG1, survival data sources and ethnicity.

#### 2.9.3 Sensitivity analysis

In order to test the stability of meta-analysis results of indicators, a one-by-one elimination method will be adopted for sensitivity analysis.

#### 2.9.4 Publication bias

If more than 10 studies were performed, funnel plots could be used to evaluate the existence of publication bias.^[21]^ Moreover, Egger and Begger test were performed for the evaluation of potential publication bias.

## 3 Bioinformatics analysis

### 3.1 The expression of TUG1 in osteosarcoma was analyzed by public database, and the survival of TUG1 was analyzed

The expression level of TUG1 in osteosarcoma and its relationship with tumor grade, sex, and race were analyzed by using UALCAN database, and the survival of TUG1 was analyzed. The survival curve was analyzed by Kaplan–Meier and statistically tested by log-rank test.

### 3.2 MicroRNA prediction of interaction with TUG1

RegRNA 2.0 biological software was used to predict the possible microRNA binding sites on TUG1 sequences. When predicting the binding site of TUG1 and microRNA, the minimum folding free energy (MFE) is ≤–20. According to the score of pairing lncRNA with its microRNA, the higher the score is, the stronger the binding ability of table lncRNA-microRNA is. At the same time, the human micoRNAs disease database (HMDD) was applied to search and analyze the microRNAs that is related to osteosarcoma and its intersection was chosen to analyze the micoRNAs that may interact with TUG1 in osteosarcoma. The expression level of micoRNA in various cancers was detected by OncomiR platform.

### 3.3 Prediction of microRNA target genes

The Targetscan network online platform and microTCDS were used to predict the target gene of micoRNA. In order to avoid too many false positive results, the prediction results of the 2 software were intersected, and the regulatory network was further analyzed.

### 3.4 Construction of lncRNA–microRNAs–mRNAs interaction network

The potential microRNAs regulated by TUG1 were selected to predict the target genes regulated by each microRNA, and Cytoscope was used to integrate TUG1-microRNAs and microRNAs-mRNAs networks, so as to form a TUG1-microRNAs-mRNAs network regulation relationship.

### 3.5 Functional analysis of microRNA target gene

The biological function of the target gene was analyzed by FunRich platform. The cellular components, molecular function, and biological process items in GeneOntology (GO) analysis and Kyoto Encyclopedia of Genes and Genomes (KEGG) pathway items in Pathways were selected and analyzed.

## 4 Discussion

Osteosarcoma is often associated with metastasis and the prognosis is generally poor. Previous tumor size and location, chemotherapy response, alkaline phosphatase level, and P-glycoprotein expression may be independent factors in judging the prognosis of osteosarcoma. However, with the in-depth study of osteosarcoma-related lncRNA, it is obvious that the abnormal expression of lncRNA in osteosarcoma is closely related to the poor prognosis of patients.^[22–24]^ As a prognostic biomarker. lncRNA is conducive to early detection, early diagnosis, and early treatment of osteosarcoma, and it helps to improve the overall survival rate of osteosarcoma patients, with important potential clinical application value.

TUG1 is not only involved in the molecular mechanism of osteosarcoma, but also closely related to the clinicopathological characteristics and prognosis of osteosarcoma. The elevated TUG1 expression indicates a poor clinical prognosis in patients with osteosarcoma, and may be a biomarker for the evaluation of the prognosis of patients with osteosarcoma. In this study, the role of TUG1 in prognosis in patients with osteosarcoma was analyzed by conducting meta-analysis and bioinformatics. However, the specific mechanism of action of TUG1 in the pathogenesis of osteosarcoma and its correlation with clinical prognosis still need to be further clarified and verified by further basic studies through more large-sample and high-quality clinical trials.

## Author contributions

**Conceptualization:** Fudong Liang, Zheng Ren.

**Data curation:** Fudong Liang, Zheng Ren, Chenyang Li.

**Formal analysis:** Chenyang Li.

**Funding acquisition:** Fudong Liang.

**Investigation:** Fudong Liang.

**Methodology:** Chenyang Li.

**Project administration:** Fudong Liang.

**Resources:** Chenyang Li.

**Software:** Yuling Gan.

**Supervision:** Yuling Gan.

**Validation:** Yuling Gan, Xiuxin Liu.

**Visualization:** Xiuxin Liu.

**Writing – original draft:** Fudong Liang, Zheng Ren.

**Writing – review & editing:** Fudong Liang, Zheng Ren.
